# Enhancing the Accuracy of Pedicle Screw Placement Using 3D-Printed Screw-Guiding Techniques in the Lumbosacral Region for Small Breed Dogs: A Cadaveric Study

**DOI:** 10.3390/ani15010014

**Published:** 2024-12-25

**Authors:** Jin-Yeong Kim, Ho-Hyun Kwak, Heung-Myong Woo, Junhyung Kim

**Affiliations:** Department of Veterinary Medicine, Kangwon National University, Chuncheon-si 24341, Gangwon-do, Republic of Korea; yeong0029@kangwon.ac.kr (J.-Y.K.); kwakhh@kangwon.ac.kr (H.-H.K.); woohm@kangwon.ac.kr (H.-M.W.)

**Keywords:** three-dimensionally-printed guide, screw guide, cannulated screw, pedicle screw, DLSS

## Abstract

A 3D-printed guide is an effective method for accurately placing pedicle screws in dog vertebrae. While a conventional drill guide allows precise pilot hole formation, it can lead to potential screw wobbling during insertion. In this study, we applied a technique that assists with both drilling and screw insertion, and we compared the accuracy of screw placement using this approach with that achieved by the conventional drill guide. The screws were divided into three groups: Group A (drill guide), Group B (cannulated guide), and Group C (screw guide). The accuracy of screw placement was assessed by comparing preoperative and postoperative CT images. Group A exhibited the largest angular deviation. Group C exhibited significantly smaller deviations in entry point, exit point, angle α, and angular deviation than Group A. In Group B, only the exit-point deviation was significantly smaller than that in Group A. Furthermore, the angular deviation in Group C was significantly smaller than that in Group B. In conclusion, 3D-printed screw-guiding techniques improved the accuracy of pedicle screw placement, with screw guides outperforming cannulated guides, making them a viable option for small breed dogs.

## Simple Summary

Degenerative lumbosacral stenosis (DLSS) can occur in small dogs for several reasons. Most 3D-printed guides used for spinal surgery in veterinary medicine are conventional guides. In this study, we used screw-guiding techniques to insert pedicle screws to minimize screw wobbling that may occur during screw insertion. We compared the accuracy of this technique with that of a conventional guide. The results showed that the screws placed using the screw-guiding technique demonstrated higher accuracy than those placed using the conventional guide.

## 1. Introduction

Degenerative lumbosacral stenosis (DLSS), commonly referred to as cauda equina syndrome or lumbosacral compression, is a prevalent cause of lumbosacral pain and pelvic limb dysfunction in dogs [1]. While DLSS is more frequently reported in medium-to-large breed dogs, it can also occur in small-breed dogs [2,3,4]. DLSS can result from various pathophysiological causes, including degenerative changes or intervertebral disc protrusion, bony or soft tissue proliferation, lumbosacral instability due to lumbosacral subluxation, discospondylitis, excessive lumbosacral motion, and congenital vertebral malformations [3,5,6]. Lumbar vertebral fractures or luxations in small breed dogs are generally caused by severe trauma, such as car accidents, when external forces overwhelm the physiological stabilizers of the spine [4]. Discospondylitis is an infection affecting the intervertebral disc and adjacent vertebral bodies, which can lead to soft tissue and bone proliferation, vertebral instability, and resorption of the subchondral bone [6,7]. When discospondylitis occurs at a single segment, it is most common at the lumbosacral junction and can cause secondary cauda equina syndrome. Subsequent spinal canal or intervertebral foramina stenosis resulting from these various causes can compress the cauda equina nerve, leading to pain, lameness, and varying degrees of neurological deficits [3,5].

Surgical treatment is indicated when conservative treatments—such as medical procedures, weight loss, and strict restriction of movement—fail to improve pain or neurological deficits or when lumbosacral instability persists [2,3,4,6]. Surgery aims to decompress the cauda equina or relieve entrapped nerve roots directly or indirectly using stabilization techniques [3,5]. The most common decompressive surgery is dorsal laminectomy, which is often combined with foraminotomy, discectomy, or facetectomy, depending on the case [1,5]. Although short-term improvements in clinical symptoms are frequently observed in patients who undergo decompression surgery, the recurrence rate of clinical symptoms remains high in the long-term follow-up [2,8]. The lumbosacral joint in dogs exhibits the greatest mobility among all lumbar vertebrae [9]. Decompression alone may exacerbate lumbosacral instability, leading to degenerative changes or the recurrence of clinical symptoms. Moreover, the dynamic instability arising from soft tissue damage associated with DLSS stressed the need for stabilization techniques [10]. Therefore, intervertebral stabilization techniques should be considered when there is a concern about postoperative instability [1,3,8,11].

Various techniques for dorsal distraction stabilization have been used, including cross-pinning [12], trans-articular facet screws [10,13], placement of pins or screws in the pedicles or vertebral bodies using polymethylmethacrylate (PMMA) [14,15,16], fixation with string of pearls (SOP) plate system [17], and pedicle screw-rod fixation (PSRF) [2,8,11,18]. These implants provide stability to the lumbosacral joint after surgery, correct pre-existing instability, and prevent the progression of degenerative changes [1,3]. However, achieving safe and accurate placement of screws or pins during dorsal fixation is challenging and poses a risk of iatrogenic injury to vital structures, including the cauda equina, nerve roots, or vasculature [4,6,8]. Several studies have reported anatomical landmarks for entry points and ideal trajectories in the lumbosacral region [8,11,19,20], but the reported rates of vertebral canal perforation with freehand screw placement range from 8.3% to 42.6% [3,8,18]. Additionally, the morphology of the vertebral bodies and pedicles varies significantly between individuals [19], and congenital malformations, such as transitional vertebrae or degenerative changes, further complicate accurate screw placement [21,22].

Various methods, including fluoroscopy-assisted and CT imaging navigation, have been developed to improve the accuracy of the freehand technique. Recently, there has been a growing interest in using three-dimensional (3D) patient-specific guides [3,6,23], and studies have demonstrated that these guides provide higher accuracy than the freehand technique. The 3D-printed guides used in previous studies were conventional drill guides that focused on creating accurate pilot holes by determining the entry point location and insertion angle to assist drilling. However, the screw placement process still relied on the freehand technique. A recent study found that even an accurately created pilot hole does not always guarantee that screws will follow the intended trajectory [24]. Deviation of the screws after placement was more significant than that immediately after pilot hole formation [25,26]. This suggests that screw wobbling can occur during the placement process, leading to misalignment from the planned trajectory [27].

To prevent screw wobbling during insertion and improve alignment, we utilize screw-guiding techniques, employing a modular 3D-printed guide to assist with both drilling and screw placement. In human medicine, cannulated screws or screw guides are used to enhance screw placement accuracy for PSRF and in the treatment of scoliosis [28,29]. To the best of our knowledge, no previous study has used screw-guiding techniques to place pedicle screws or compared the accuracy of pedicle screw placement using these techniques with conventional drill guides in veterinary medicine. Therefore, this study aimed to evaluate and compare the accuracy of pedicle screw placement using cannulated screws and screw guides with that using conventional drill guides. Additionally, we aimed to assess the reproducibility of screw wobbling in canine vertebrae when using conventional drill guides and to evaluate the efficacy of pedicle screw placement in L6 vertebral segments.

## 2. Materials and Methods

### 2.1. Cadaveric Study

In this study, the lumbar spine (L6, L7) and sacrum (S1) of six small-breed dogs weighing < 10 kg (median: 5 kg, range: 2.5–9.2 kg) were used. The breeds included Beagles and mixed breeds, and all dogs were euthanized for reasons unrelated to the present study. Vertebral segments with degenerative changes were included, while those with spinal fractures or tumors were excluded. The cadavers were stored at −20 °C and thawed at room temperature 24 h before surgery. All the procedures were approved by the Institutional Animal Care and Use Committee of Kangwon National University (Approval No. KW-240502-1).

### 2.2. Study Design

**Figure 1:**
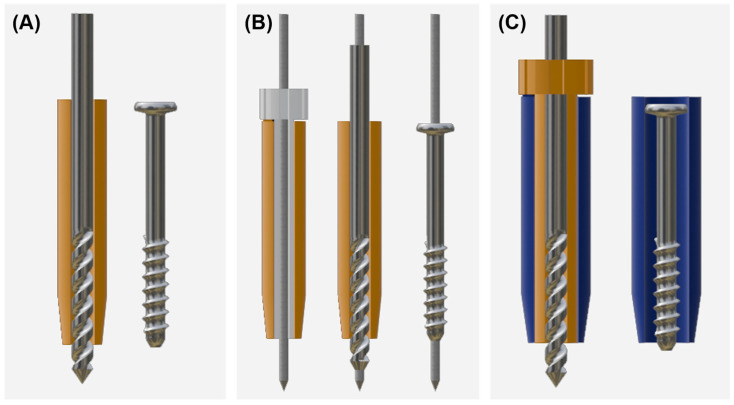
Schematic image of each group. Each group is represented as (**A**) drill guide, (**B**) cannulated guide, and (**C**) screw guide. The orange object is a drill guide, the white one is a pin guide, and the blue one is a screw guide. The metallic objects are a drill bit (or cannulated drill bit), a guide pin, and a cannulated screw, respectively.

Two self-tapping cannulated screws (2.3 or 3.0 mm, ARIX, JEIL MEDICAL, Seoul, Republic of Korea) were placed in each vertebral segment, for a total of 36 screws. The screws were divided into three groups depending on the screw placement method: (A) drill guide group (*n* = 12) that used conventional drill guides, (B) cannulated guide group (*n* = 12) with a detachable pin guide inside the drill guide, and (C) screw guide group (*n* = 12) that had a detachable drill guide inside the screw guide. A schematic representation of each group is illustrated (Figure 1). The type of screw placement to be used on each side of the vertebral segments was determined through block randomization for each cadaver. To ensure even distribution, each block contained two screws from each group, ensuring that no identical groups were present in a single vertebral segment.

### 2.3. Surgical Planning

**Figure 2:**
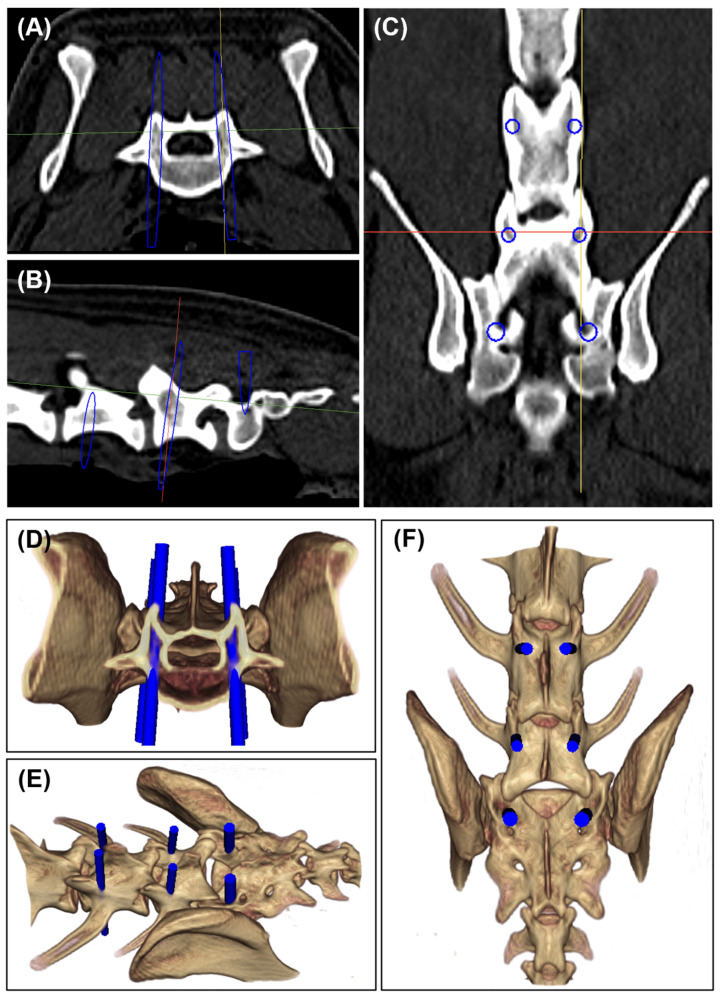
Three-dimensional images showing the entry point and insertion angle of the screw. (**A**–**C**) Multiplanar reconstruction images showing cross sections in the axial plane (**A**), sagittal plane (**B**), and coronal plane (**C**). (**D**–**F**) Volume rendering images showing the cranial (**D**), dorsolateral (**E**), and dorsal (**F**) views. The blue outline and cylindrical objects represent the screws with ideal trajectories.

Preoperative multi-slice computed tomography (CT) images of the lumbosacral region of the dog cadavers were obtained using a 16-slice helical CT scanner (Alexion, Canon, Japan) with a 0.5 mm slice thickness. The images were saved in the Digital Imaging and Communications in Medicine (DICOM) format and imported into medical imaging software (3D slicer version 5.6.2, accessed on 5 April 2024, www.slicer.org ) to create vertebral models and 3D surgical planning. We determined the entry points and ideal screw trajectories for L6, L7, and S1 through volume rendering and multiplanar reconstruction (MPR) of the images (Figure 2). These entry points and trajectories were informed by previous studies [11,19,30]. Briefly, the L7 entry point was defined as the intersection of the vertical line adjacent to the caudal border of the cranial articular process of L7 and the horizontal line bisecting the transverse process. The L6 entry point was set similarly. For S1, the entry point was identified at the intersection of the vertical line adjacent to the caudal border of the cranial articular process of S1 and the horizontal line bisecting the caudal border of the cranial articular process of S1 and the cranial border of the intermediate sacral crest. Ideal screw trajectories for each vertebral segment were planned to avoid damaging important structures located around the vertebra, such as the cauda equina, nerve roots, and blood vessels (vertebral artery and veins, common iliac artery, and external iliac artery). To determine the screw trajectory, angle α and β were used and their definitions were consistent with those in previous study [3]. On the axial plane, the mediolateral angle between the sagittal plane and the screw trajectory, defined as angle α, was constrained to a deviation of 0 to approximately 15˚ [19,30]. On the sagittal plane, the craniocaudal angle between the plane passing through the cranial vertebral endplate and the screw trajectory, defined as angle β, should also be maintained within the range of 0 to approximately 15˚. However, depending on the morphology of the vertebra, the angle β was adjusted to maximize bone purchase of the screw without damaging the cranial vertebral endplate. After the screw trajectory was determined for all the vertebral segments, the 3D coordinates (R, A, and S) of the entry and exit points of the screws were measured. Additionally, the pedicle width, screw length, and angle α were obtained from the axial plane of the corresponding MPR image, while angle β was measured on the sagittal plane. The screw size was selected to ensure it did not exceed 70% of the pedicle width, while the screw length was determined to maximize the positioning of the threaded portion within the cancellous bone of the vertebra, allowing for bi-cortical fixation.

### 2.4. Design and Fabrication of 3D-Printed Guide

**Figure 3:**
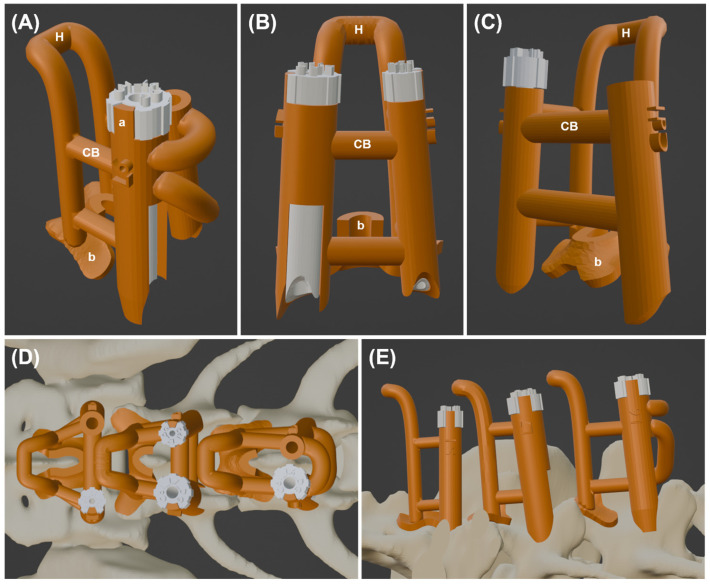
Design and structure of the guide are illustrated using 3D modeling software (Blender). The orange structure represents the outer guide with guide body, and the white structure represents the inner guide that fits inside the outer guide. The guide for L6 is designed to accommodate a screw guide on the right side and a drill guide on the left side (**A**). The guide for L7 features a screw guide on the right side and a cannulated guide on the left side (**B**). For S1, the design includes a cannulated guide on the right side and a drill guide on the left side (**C**). The connecting bar between the outer guides is either straight or curved, depending on the position of the spinous process. Guides applied to 3D-reconstructed vertebra at dorsal view (**D**) and lateral view (**E**). a, anchor; H, handle; CB, connecting bar; b, base of guide.

**Figure 4:**
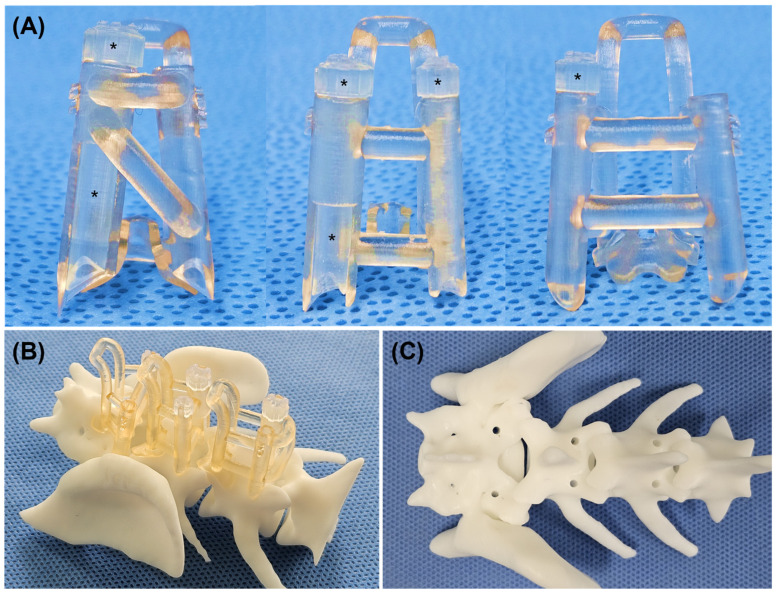
Three-dimensionally-printed guide and vertebra model. The guide depicted in Figure 3 was 3D printed and is shown from the cranial view (**A**). The guide body, comprising the outer guide, handle, base, and connecting bar, was printed using BioMed Amber resin, while the inner guide (*, Black asterisk) was printed using BioMed Clear resin. Rehearsal surgery was performed using the vertebral model and guide; the guide was applied to the vertebra (**B**); after drilling (**C**).

The stereolithography (STL) file of the vertebral model was exported from the medical imaging software to the 3D modeling software (Meshmixer, Autodesk, San Rafael, CA, USA) (Blender, Blender Foundation, Amsterdam, Netherlands), and the vertebra was reconstructed three-dimensionally. The base of the guide was set in contact with the surface of the 3D vertebral model with a thickness of 1.25–1.5 mm. For L6 and L7, it was designed to anchor to the dorsal surface of the caudal lamina and caudal part of the spinous process. For S1, it was designed to anchor to the dorsal surface of the cranial lamina and cranial part of the median sacral crest to minimize displacement in the craniocaudal direction. A handle with a thickness of 3–4 mm was added to the base to make a guide easier to manipulate and ensure full adherence to the dorsal surface of the vertebra by applying a vertical force. After that, a cylinder with a length of 15–20 mm was created and placed at the coordinate of the previously measured entry point, and the angles α and β were reflected to have an accurate trajectory. The shape of the cylinder and the presence of an inner guide varied depending on the group. In Group A, a single drill guide was designed to fit the drill bit (1.8 or 2.2 mm) depending on the size of the screw. In Groups B and C, a detachable inner guide was added to adopt a modular system. For Group B, the inner guide was designed to fit the guide pin (0.8 or 1.1 mm), and the outer guide was designed to fit the drill bit. In Group C, the inner guide was designed to fit the drill bit, and the outer guide was designed to fit the screw head (3.5 or 4.5 mm) to ensure that the screw head was inserted along the guide. The screw guide formed a rectangular defect with the same height as the length of the screw remaining in the cranial part of the guide, which served as an indicator and facilitated screw removal. The thickness of the outer guide was 1–1.25 mm, and the thickness of the inner guide was adjusted to fit the outer guide. All guides, including the outer and inner guides, were designed to contact the surface of the 3D vertebral model. To position the inner guide accurately and prevent it from rotating out of place, an anchor was fixed to the upper part of the outer guide. Additionally, several bars were used to connect each outer guide and link the outer guide to the handle, thereby enhancing overall stability (Figure 3). All the guides were printed using a stereolithography apparatus (SLA) 3D printer (Form 3B+, Formlabs, Somerville, MA, USA) with two medical-grade biocompatible photopolymer resins (BioMed Clear resin and BioMed Amber resin, Formlabs, Somerville, MA, USA) (Figure 4A). After post-processing, the guide was steam-sterilized. Additionally, to understand the anatomical shape and assess the suitability of the guide for installation, a 3D vertebral model was printed using another SLA 3D printer (Phrozen Mega 8 K, Phrozen, Hsinchu, Taiwan). Rehearsal surgery was performed using a 3D printed vertebral model and guides in all cadavers (Figure 4B,C).

### 2.5. Surgical Procedure

**Figure 5:**
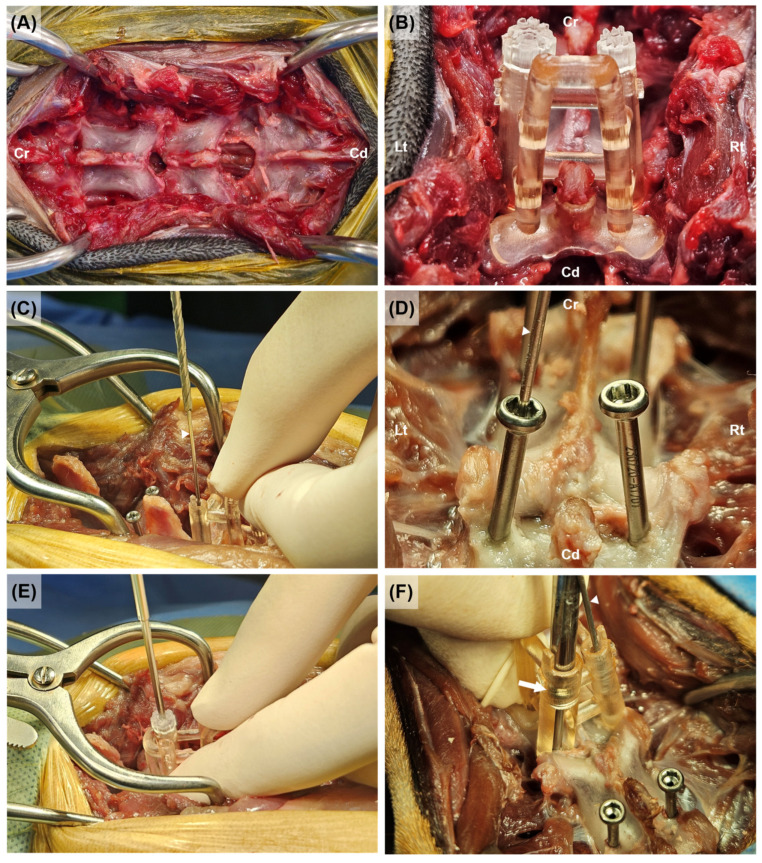
The screw placement using the guides of each group. The soft tissue and periosteum around the spinous processes and dorsal surface of the lamina from L6 to S1 were removed to allow the guide to adhere tightly (**A**). The guide was positioned on the L7 vertebral body, with the left side for the screw guide and the right side for the cannulated guide (**B**). For the cannulated guide, after inserting the guide pin (White arrowhead) along the inner pin guide, drilling was performed using a cannulated drill bit (**C**). The cannulated screw on the left was inserted along the guide pin (**D**). For the screw guide, drilling was performed using a normal drill bit along the inner drill guide (**E**). After removing the inner guide, the screw head (white arrow) was inserted along the screw guide (**F**). Cr, Cranial; Cd, Caudal; Lt, Left; Rt, Right.

All dogs were placed in the sternal recumbent position with their hind limbs drawn cranially. Using the iliac wing and spinous process of L6 as landmarks, a midline incision was made from the level of L5 to the caudal part of the median sacral crest of the sacrum. The superficial and deep fascia of the lumbosacral region were incised, the epaxial muscles and ligaments attached to the spinous process were separated, and the soft tissues around the articular process were elevated if necessary. To allow the guide to adhere tightly to the vertebrae, the soft tissue and periosteum were sufficiently removed around the dorsal surface of the lamina and spinous processes (Figure 5A). With the inner guide mounted on the outer guide, the surgeon held the handle of the guide with the non-dominant hand and positioned the guide in the desired location (Figure 5B). In Group A, drilling was performed along the drill guide to form a pilot hole of an appropriate size, the guide was removed, and the screw was inserted freehand. In Group B, the guide pin was inserted along the inner guide. Subsequently, the pin guide was removed, and a pilot hole was created along the drill guide using a cannulated drill bit while avoiding penetrating the far cortex (Figure 5C). After removing the guide, the screw was placed under the aid of the guide pin (Figure 5D). In Group C, the pilot hole was formed along the inner guide (Figure 5E), which served as a drill guide. After removing the drill guide, the screw was inserted under the aid of the screw guide (Figure 5F). Drilling was performed in random order for each vertebra; however, when Group C was included, the screws of Group C were placed first because of the structural characteristics of the guide, followed by the screws on the opposite side.

### 2.6. Outcome Measurement

**Figure 6:**
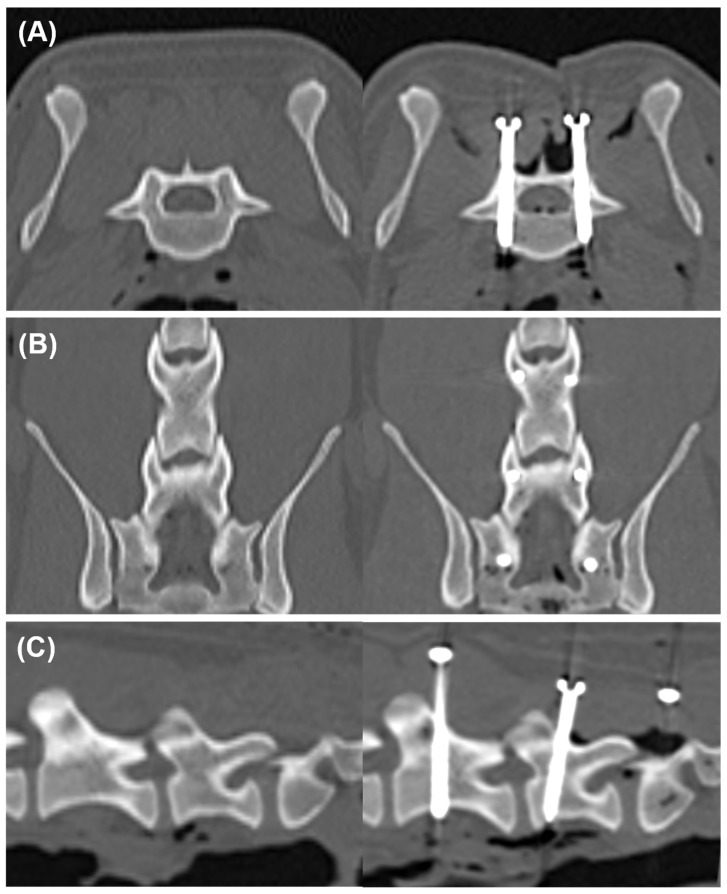
Preoperative and postoperative CT images obtained by multiplanar reconstruction (MPR). Each image was superimposed to measure deviation on the axial plane (**A**), coronal plane (**B**), and sagittal plane (**C**).

**Figure 7:**
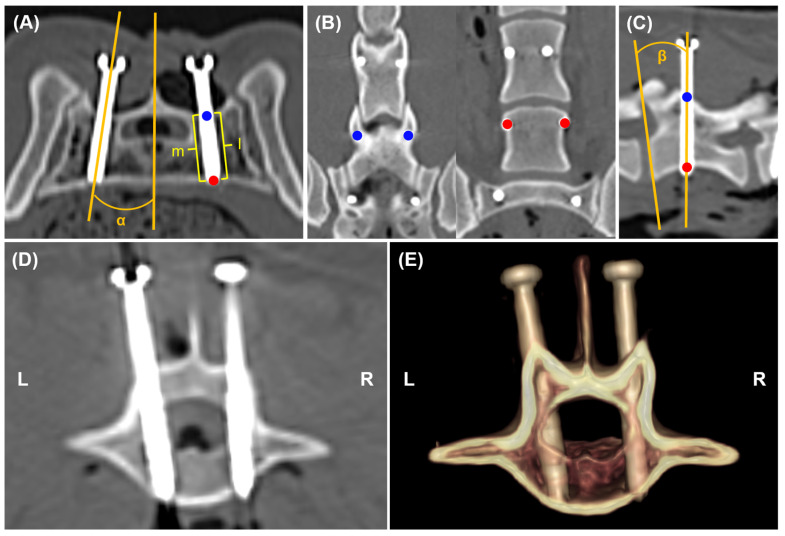
Outcome measurement with postoperative CT images. The entry point (blue circles) and exit point (red circles) were determined at the point where the screw meets the cortical bone on the axial plane (**A**), coronal plane (**B**), and sagittal plane (**C**). Angle α was defined as the mediolateral angle between the sagittal plane and the screw trajectory (**A**). Angle β was defined as the craniocaudal angle between the plane passing through the cranial vertebral endplate and the screw trajectory (**C**). Medial and lateral bone stock refers to the lengths of the medial (m) and lateral (l) screw lines surrounded by bone (**A**). Grade of vertebral canal breach was evaluated on the axial plane (**D**) and volume rendering image (**E**). Screw on the right indicates Grade 1 breach, while screw on the left indicates Grade 2 breach. L, Left; R, Right.

**Figure 8:**
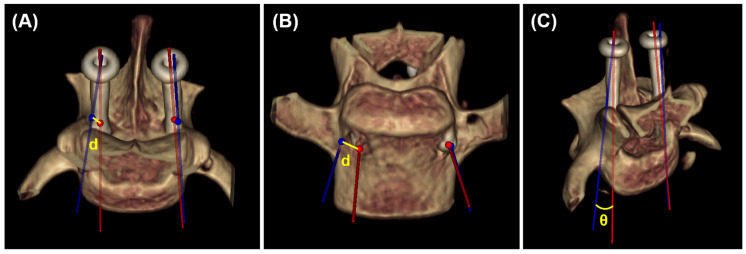
The deviation between preoperative planned trajectory and the postoperative screw trajectory. Deviation was assessed using volume rendering images and the coordinates of the entry and exit points. The blue line and circles represent the planned trajectory along with the entry and exit points, while the red line and circles represent the screw trajectory, including the corresponding entry and exit points. The entry point deviation was measured by calculating the distance (d) between the two circles located on the dorsal surface of the vertebral body (**A**). The exit point deviation was similarly determined by measuring the distance (d) between the two circles located on the ventral surface of the vertebral body (**B**). The angular deviation was calculated by measuring the three-dimensional angle (θ) between the two lines (**C**).

A postoperative CT scan was performed, and the DICOM file was transferred to medical imaging software for the evaluation of screw placement by a veterinarian. To measure the degree of deviation from the planned screw trajectory, the postoperative image was superimposed on the preoperative image for each vertebral segment using a method similar to that used in a previous study [4]. The alignment of the two images was adjusted using volume rendering. Subsequently, landmarks were used to obtain superimposed MPR images (Figure 6). The landmarks included the spinous and transverse processes at L6 and L7, as well as the intermediate sacral crest and medial sacral crest at S1. In the postoperative MPR image, we measured the three-dimensional coordinates of the entry and exit points, screw insertion angles α and β, medial and lateral bone stocks, and grade of vertebral canal breach (Figure 7). The bone stock and vertebral canal breach were measured as described previously [3]. Grade 1 indicates a breach of the medial pedicle smaller than the diameter of a single screw, while Grade 2 indicates a breach of the medial pedicle that exceeds the diameter of a single screw. Subsequently, we measured the deviation of the entry point, exit point, angles α and β, and the angular deviation, which is the angle between the planned screw trajectory and the postoperative screw trajectory (Figure 8).

### 2.7. Statistical Analysis

Statistical analysis was performed using SPSS version 29.0 (IBM, Armonk, NY, USA), and statistical significance was set at *p*-value < 0.05. The distribution of continuous variables was presented using the mean ± standard deviation. All variables were assessed for normal distribution using the Kolmogorov–Smirnov or Shapiro–Wilk normality test. Among the variables, entry point deviation, exit point deviation, angular deviation, medial bone stock, and lateral bone stock satisfied normality; therefore, analysis of variance (ANOVA) was performed. A one-way ANOVA was performed on variables that satisfied both normality and equal variance, and the Tukey HSD test was used as a post hoc test. Welch’s ANOVA was performed on variables that satisfied normality but did not satisfy equal variance, and the Games–Howell test was used as a post hoc test. The Kruskal–Wallis and Mann–Whitney U tests were used as non-parametric tests, and the Bonferroni correction method was used as a post hoc test. A Fisher’s exact test was performed to compare the percentages of vertebral canal breaches among the different groups and across each vertebral segment.

## 3. Results

### 3.1. Entry Point and Exit Point

**Figure 9:**
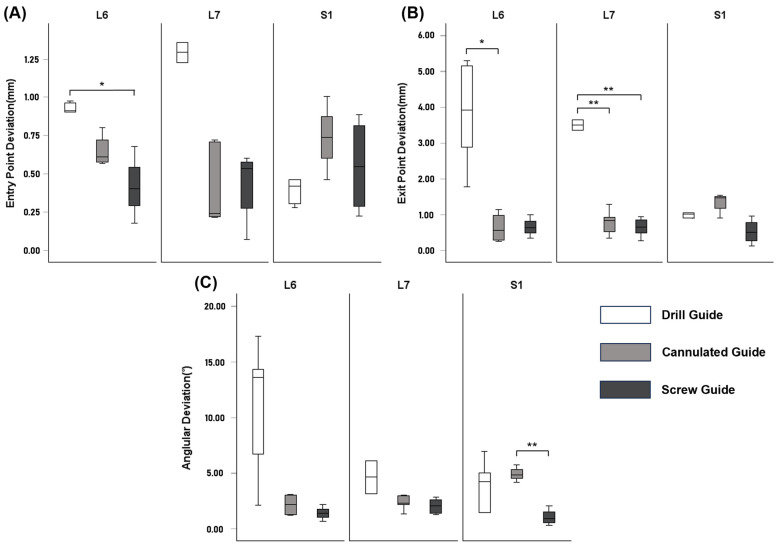
Box plot of entry point deviation (**A**), exit point deviation (**B**), and angular deviation (**C**). The result’s significance is indicated by the symbol (*), where one symbol corresponds to a *p*-value of 0.01 < *p* ≤ 0.05, and two symbols indicate a *p*-value of *p* ≤ 0.01.

In this study, a total of 36 screws were inserted in 18 vertebral segments, and the preoperatively planned trajectory was compared with the actual screw trajectory in 3D. The mean entry point deviation for all screws was 0.613 ± 0.324 mm, and the mean exit point deviation was 1.4 ± 1.359 mm (Table 1). In Group A, the exit point deviation was significantly larger than the entry point deviation (*p* = 0.002). In Groups B and C, no significant differences were observed between the entry point and exit point deviations. The entry point deviation in Group A (drill guide) was 0.804 ± 0.379 mm, which was significantly larger than that in Group C (screw guide) (*p* = 0.022). In contrast, no significant difference was observed between Group A and Group B or between Group B (cannulated guide) and Group C. The exit point deviation in Group A was 2.727 ± 1.637 mm, which was significantly larger than in Group B (*p* = 0.006) and Group C (*p* = 0.002), with no significant difference between Group B and Group C. Group C demonstrated the smallest deviation for both the entry and exit points. No significant differences were observed in the entry or exit point deviations between the vertebral bodies. The accuracy of screw placement based on the screw placement method for each vertebral body was illustrated using a box plot (Figure 9). In the L6 segment, the entry point deviation in Group A was significantly larger than that in Group C (*p* = 0.021), and the exit point deviation in Group A was significantly larger than in Group B (*p* = 0.016) and Group C (*p* = 0.017). In the L7 segment, the exit point deviation in Group A was significantly larger than that in Group B (*p* < 0.001) and Group C (*p* < 0.001).

### 3.2. Screw Insertion Angle

For all screws, the mean angle α deviation was 2.632 ± 4.085°, the mean angle β deviation was 1.825 ± 1.624°, and the mean angular deviation was 3.793 ± 3.886° (Table 2). In Group A, angle α deviation was significantly larger than angle β deviation (*p* = 0.039). In Group B, no significant difference was observed between angle α deviation and angle β deviation, and in Group C, angle β deviation was significantly greater than angle α deviation (*p* = 0.012). Angle α deviation in Group A was 5.896 ± 1.678°, which was significantly larger than that in Group C (*p* < 0.001). In contrast, no significant difference was observed between Group A and Group B or between Group B and Group C. No significant differences were observed in angle β deviation between the groups. Group C exhibited the smallest angular deviation at 1.56 ± 0.801°, which was significantly smaller than that of Group A (*p* = 0.014) and Group B (*p* = 0.024). No significant difference was observed in angular deviation between Group A and Group B. No significant differences in angular deviation were observed between the vertebral bodies. In the S1 segment, the angular deviation in Group B was significantly larger than that in Group C (*p* = 0.004) (Figure 9).

### 3.3. Bone Stock

The mean medial bone stock for all screws was 12.396 ± 2.133 mm, and the mean lateral bone stock was 11.782 ± 2.320 mm (Table 3). No significant differences were observed in medial bone stock (*p* = 0.361) or lateral bone stock (*p* = 0.250) between the groups. The medial bone stock was largest in Group B, measuring 12.821 ± 2.226 mm, while the lateral bone stock was largest in Group A, measuring 12.277 ± 2.215 mm.

### 3.4. Vertebral Canal Breach

No significant differences were observed in vertebral canal breach between the groups (*p* = 0.281) (Table 4). In Group A, breaches were observed in 50% of the screws, with Grade 2 breaches occurring exclusively in Group A. Group B had the lowest incidence of vertebral canal breach at 16.7%. The occurrence of vertebral canal breaches was significantly higher in the L6 segment (75%) than in the L7 (16.7%) and S1 (0%) segments (*p* < 0.001).

## 4. Discussion

This study compared the accuracy of pedicle screw placement in the lumbosacral region of small dogs using a screw-guiding technique and conventional drill guides. The results indicated significantly higher accuracy in the screw guide group for all measurements except for angle β, bone stock, and vertebral canal breach. Group A exhibited a significantly larger deviation at the exit point than at the entry point and showed the largest angular deviation. In contrast, Groups B and C demonstrated no significant difference between the entry and exit points, with relatively small angular deviations. This indicates that, although the starting point of screw insertion was accurate when using the conventional drill guide, screw wobbling occurred during screw placement, resulting in a deviation from the planned trajectory. In conclusion, the screw-guiding technique improved the accuracy of screw placement, with screw guides yielding better results than cannulated guides. 

Previous veterinary studies on 3D-printed guides for pedicle screw placement have primarily used conventional drill guides. A recent study suggested that mediolateral loading during drilling led to greater deviations at the exit point than at the entry point [4]. However, a study in human medicine found that screws placed using a guide exhibited greater deviations compared to a pilot hole. This suggests that even if a pilot hole was created accurately, the screw may deviate from the planned trajectory [24]. Screw wobbling is believed to result from a lack of alignment support during screw placement, indicating the need for assistance with screw insertion. Screw wobbling can be more noticeable with polyaxial or monoaxial pedicle screws than with regular screws, as the center of gravity of a pedicle screw is located in the tulip head where the rod is attached. This increases the chance of misalignment during freehand screw insertion. These issues can be resolved by improving the alignment with a screw-guiding technique to assist in screw insertion.

Placing screws into the vertebra without screw guidance increases the likelihood of screw loosening, which is one of the most common complications of spinal fixation surgery in human medicine [31,32]. Screw loosening is influenced by the bone quality of the vertebra and screw placement technique. A study comparing freehand placement to power-assisted placement in pedicle screws revealed a greater degree of screw wobbling during freehand placement [27]. Changing the grip during freehand screw placement can lead to misalignment, causing torque to be applied in the wrong direction and resulting in screw wobbling. Conversely, power-assisted insertion prevents this problem, ensuring accurate screw placement. Likewise, screw-guiding techniques may help reduce screw wobbling, as they prevent deviation from the planned trajectory, even when inserted freehand, resulting in a reduced incidence of screw loosening.

Considering the anatomical and physiological characteristics of the canine vertebrae, assistance in screw insertion may be more important in veterinary medicine than in human medicine. In general, the ratio of cortical to trabecular bone in dogs is approximately 80:20 for long bones and 65:35 for vertebrae [33]. Dogs have bone characteristics most similar to humans [34], but they generally have a higher proportion of trabecular bone than cortical bone compared to humans [35,36]. Additionally, the trabecular bone in dogs has a lower bone density and modulus than that in humans [37], and a comparison with non-human primates showed that the lumbar vertebrae in dogs have a higher trabecular density but lower thickness than those in primates [38]. Considering these characteristics, the potential for screw wobbling is thought to be higher in dogs than in humans. Furthermore, dog vertebrae are generally smaller than those of adult humans [39], so even minor displacements can have a relatively large impact on the outcomes. Accurate screw placement is especially crucial in small breed dogs compared to large breeds, due to the deepened degree of this.

Representative screw-guiding techniques include methods that use a cannulated system with a guide pin or a screw guide that directly contacts the screw head. In this study, both methods were applied in a modular form with an attachable inner guide. Both the cannulated screw and screw guide enabled more accurate pedicle screw placement than the conventional drill guide. However, differences in accuracy were observed between the screw-guiding techniques. Group C demonstrated better results than those of Group A in most measurements and significantly outperformed Group B in terms of angular deviation. In contrast, Group B demonstrated significantly better results than those of Group A only in terms of the deviation of the exit point. This may be attributed to the small thickness of the guide pin, which does not provide sufficient strength or stiffness based on the area moment of inertia (AMI) [40]. As a result, displacement can occur due to loading during drilling or torque applied during screw insertion. If a larger screw is used and the guide pin thickness increases, the installation accuracy in Group B may change. However, the cannulated system has the potential to cause additional injuries, as the guide pin must be installed before inserting the screw. Additionally, it has limitations regarding the variety of sizes available compared to regular screws. In contrast, a screw guide does not require an additional implant. The inner diameter of the screw guide can be adjusted to match the diameter of the screw head regardless of the type of screw used. This reduces the risk of further damage and minimizes restrictions.

Neuronavigation can be used to assist in screw placement by providing real-time 3D images based on MRI or CT imaging data. This technology can accurately identify the nervous structures during surgery, making the procedure safer and more precise. When combined with 3D rotational fluoroscopy, such as the O-arm, intraoperative 3D images can be obtained, significantly improving the accuracy of screw placement in spinal surgery. This allows for real-time confirmation of the screw’s location and insertion [41]. However, these devices are generally expensive, difficult to operate, and can increase radiation exposure and surgery time due to additional procedures, such as intraoperative imaging and fiducial-based registration. In contrast, the screw-guiding technique is intuitive and cost-effective, as it requires only precise implant placement using a guide based on the preoperative CT image, thereby reducing both radiation exposure and surgical time. In the future, follow-up studies should be conducted to compare the accuracy and clinical significance of pedicle screw placement using neuronavigation and screw placement using the screw-guiding technique.

Pedicle screws can be applied to all vertebral segments in humans because the vertebral body is large and the pedicle is wide. However, in dogs, pedicle screws are primarily applied to the lumbosacral junction, where the pedicles are relatively wide, owing to their smaller vertebral bodies and narrower pedicles compared to humans [39]. However, a recent study reported that a pedicle screw was successfully installed at L6 through a dorsal approach, although the thickness of the pedicle at L6 was smaller than that at L7 [3,30]. In this study, the accuracy of screw placement was compared between each vertebral segment (L6 to S1) to assess the efficacy of pedicle screw placement at L6. No statistically significant differences were observed between the vertebral segments in any of the measurements, except for the vertebral canal breach. At L6, 9 out of 12 screws (75%) exhibited Grade 1 or 2 breaches, while at L7, only 16.7% of screws had Grade 1 or 2 breaches. In contrast, none of the screws at the S1 level demonstrated a vertebral canal breach. This observation may be attributed to the relatively narrow pedicle of L6, as in most cases, 70% of the pedicle width was close to the screw size of 2.3 mm used in this experiment. Regarding the differences between the groups by vertebral segments, Group A demonstrated significantly greater deviations in the entry and exit points than those of Group C in the L6. Additionally, Group A had greater exit point deviations than those of Group B. As shown in the box plot (Figure 9), there was no significant difference in accuracy between the vertebral segments in all measurements in Groups B and C, where the screw-guiding technique was applied. In summary, using an appropriately sized screw for L6 allows accurate insertion, similar to L7 and S1, and employing the screw-guiding technique can further enhance screw placement accuracy.

The average entry point deviation for all screws was 0.61 mm, while the average exit point deviation was 1.4 mm, with the exit point deviation being significantly greater than that of the entry point. In a previous study using a conventional drill guide to insert pedicle screws in the lumbosacral region, the entry point deviation was 1.5 mm, and the exit point deviation was 2.57 mm in the cadaver model. In clinical cases, the entry point deviation was 1.95 mm, and the exit point deviation was 2.91 mm [4]. Another study that applied the same method to the thoracolumbar region reported an entry point deviation of 0.81 mm and an exit point deviation of 0.94 mm in the cadaver model, while in clinical cases, the entry point deviation was 0.85 mm, and the exit point deviation was 1.47 mm [23]. In this study, the entry point deviation of Group A using the conventional drill guide was 0.8 mm, and the exit point deviation was 2.72 mm. In a human medicine study, pedicle screws were placed in all vertebrae using the screw-guiding method, entry point deviations of 2.36 mm and 0.97 mm were shown in the group using cannulated screws and the group using screw guides, respectively [24]. In this study, the entry-point deviations were 0.57 mm in the cannulated screw group and 0.46 mm in the screw guide group. Compared to previous studies, the deviation measurements obtained in this study are slightly lower or similar. Additionally, the fact that the exit point deviation was greater than the entry point deviation only in the conventional drill guide group suggests that screw wobbling may occur during freehand screw insertion.

The average of angle α, angle β, and the angular deviation of all screws was 2.6°, 1.82°, and 3.79°, respectively. In a prior study that inserted pedicle screws in the lumbosacral region of a cadaver model using a conventional drill guide, the deviation of angle α was generally greater than that of angle β [3]. In this study, a comparison of accuracy between groups revealed that Group A exhibited significantly higher values in angle α and angular deviation than those of Group C, whereas no differences were observed in angle β deviation. No statistically significant difference was observed between Groups A and B for any of the angle measurements. In Group A, angle α deviation was significantly greater than angle β deviation, but there was no significant difference in Group B. Conversely, angle β deviation was greater than angle α deviation in Group C. Considering that the mean of angle α deviation in Group A was 5.9°, this indicates that the angular displacement in the mediolateral direction was relatively greater than that in the craniocaudal direction, especially in the group that used the conventional drill guide. Although the angle β deviation was greater than the angle α deviation in Group C, it is difficult to interpret this as a large angular displacement in a specific direction because both values were lower than the means of other groups. This difference in angular displacement in specific directions suggests insufficient bone purchase in the mediolateral direction compared to the craniocaudal direction during screw insertion, increasing the likelihood of screw wobbling during freehand insertion. Using a screw-guiding technique can prevent this wobbling and reduce the angular deviation.

This study has a few limitations. First, the study used cadaveric specimens and had a small sample size. Additionally, the experiment did not use monoaxial or polyaxial pedicle screws for PSRF. At the time of the experiment, pedicle screws specifically designed for small-breed dogs were not readily available. Additionally, cannulated pedicle screws measuring 3.0 mm or smaller were extremely rare and challenging to use in pediatric implants for humans. Instead, regular cannulated screws were used across all groups to minimize errors between experimental groups. Additionally, the smallest cannulated screw size that could be used in the author’s situation was 2.3 mm. In toy breed cadavers weighing less than 3 kg, 70% of the pedicle width of L6 and L7 was measured to be close to 2.3 mm, suggesting that using smaller screws may be more appropriate. Using pedicle screws or smaller cannulated screws in future experiments could provide a more precise comparison between conventional drill guides and screw-guiding techniques. Another concern is that the titanium screw used in this study may have affected the accuracy evaluation of the screw owing to potential artifacts on the CT images. Furthermore, all measurements related to the accuracy of screw placement were evaluated by a single veterinarian without cross-validation.

Three-dimensionally-printed surgical guides necessitate preoperative CT scans, 3D modeling, and 3D printing, which increases the surgical planning time. Additionally, soft tissues must be removed to ensure accurate positioning of the guide at the contact base. Despite these challenges, such guides have been actively used in various orthopedic and neurosurgical procedures in veterinary medicine recently. In this study, a screw-guiding technique was applied to prevent the deviation caused by screw wobbling, which can occur when using a conventional drill guide during screw placement. Cannulated screws and screw guides exhibited higher screw placement accuracy than that of the conventional drill guide. Although pedicle screws can be successfully applied to the L6 vertebral segment, the pedicle is relatively thin, which increases the possibility of a breach. Therefore, when placing a pedicle screw at L6, it may be more suitable to choose a smaller size compared to the implant used for L7 or S1. As a result, it is crucial to select an appropriately sized screw and additionally apply a screw-guiding technique.

## 5. Conclusions

This study used cadaver specimens to evaluate the accuracy of screw placement using a screw-guiding technique. The study concluded that the insertion of pedicle screws in the lumbosacral region of small dogs using a technique to assist screw insertion can significantly improve placement accuracy compared to using the conventional drill guide. Additionally, using a modular screw guide was more effective than using a cannulated screw in preventing iatrogenic injury and improving accuracy. Therefore, using a screw guide for pedicle screw placement in small dogs is a reasonable alternative to enhance the accuracy of screw placement.

## Figures and Tables

**Table 1:** Comparison of entry point and exit point deviation between each group.

	Deviation of Entry Point (mm)			Deviation of Exit Point (mm)			
	Mean ± SD	F	*p* Value		Mean ± SD	F	*p* Value
Group A	0.804 ^a^ ± 0.379	4.113	<0.025 ^†^		2.727 ^c^ ± 1.637	10.190	<0.001 ^‡^
Group B	0.575 ^ab^ ± 0.249				0.865 ^d^ ± 0.439		
Group C	0.460 ^b^ ± 0.248				0.609 ^d^ ± 0.287		
Total	0.613 ± 0.324				1.4 ± 1.359		

†: One way ANOVA test, ‡: Welch’s ANOVA test. a, b: Tukey post hoc test (same letters indicate no significant difference). c, d: Games–Howell post hoc test (same letters indicate no significant difference).

**Table 2:** Comparison of screw insertion angle.

	Angle α Deviation (°)			Angle β Deviation (°)			Angular Deviation (°)		
	Mean ± SD	F	*p* Value	Mean ± SD	F	*p* Value	Mean ± SD	F	*p* Value
Group A	5.896 ^a^ ± 1.678	14.569	<0.001 ^†^	1.944 ± 0.625	2.665	0.264 ^†^	6.878 ^c^ ± 5.363	9.064	0.002 ^‡^
Group B	1.427 ^ab^ ± 0.310			2.226 ± 0.454			2.942 ^c^ ± 1.422		
Group C	0.573 ^b^ ± 0.166			1.304 ± 0.247			1.560 ^d^ ± 0.801		
Total	2.632 ± 4.085			1.825 ± 1.624			3.793 ± 3.886		

†: Kruskal–Wallis test, ‡: Welch’s ANOVA test. a, b: Bonferroni correction method (same letters indicate no significant difference). c, d: Games–Howell post hoc test (same letters indicate no significant difference).

**Table 3:** Comparison of bone stock.

	Medial Bone Stock (mm)			Deviation of Exit point (mm)			
	Mean ± SD	F	*p* Value		Mean ± SD	F	*p* Value
Group A	11.671 ± 1.899	1.052	0.361		12.277 ± 2.215	1.447	0.250
Group B	12.821 ± 2.226				10.864 ± 2.356		
Group C	12.696 ± 2.246				12.206 ± 2.299		
Total	12.396 ± 2.133				11.782 ± 2.320		

**Table 4:** Comparison of vertebral canal breaches.

	Grade of Vertebral Canal Breach			
Vertebra	Group A	Group B	Group C	*p* Value
Total (*n* = 36)	Grade 0–6 (50%) Grade 1–4 (33.3%) Grade 2–2 (16.7%) Breach: 6 (50%)	Grade 0–10 (83.3%) Grade 1–2 (16.7%) Grade 2–0 (0%) Breach: 2 (16.7%)	Grade 0–9 (75%) Grade 1–3 (25%) Grade 2–0 (0%) Breach: 3 (25%)	0.281

## Data Availability

All the data are discussed in the manuscript.
